# Esthetic and Functional Rehabilitation: Retreatment of Anterior Fixed Dental Prothesis With Biologically Oriented Preparation Technique and Digital Workflow

**DOI:** 10.1111/jerd.13393

**Published:** 2024-12-20

**Authors:** Belén Morón‐Conejo, Alfonso Gil, Mónica Bonfanti‐Gris, Maria Paz Salido, Francisco Martínez‐Rus

**Affiliations:** ^1^ Analysis of Techniques, Material and Instruments Applied to Digital Dentistry and CAD/CAM Procedures Research Group University Complutense of Madrid Madrid Spain; ^2^ Associate Faculty at University International of Catalunya and University of Southern California Los Angeles California USA

## Abstract

**Objective:**

This clinical case describes a multidisciplinary retreatment of a patient with anterior fixed dental prostheses (FDPs) using minimally invasive restorations and a biologically oriented preparation technique (BOPT).

**Clinical Considerations:**

A 56‐year‐old female patient, treated 30 years ago with a metal‐ceramic FDP due to dental agenesis, presented a misfit prosthesis at the gingival margin, black spaces, and food retention at the pontics. Notably, tooth number 2.6 was absent, and she exhibited a left crossbite. Her chief complaint was the compromised esthetics of her restorations. Given her coagulation disorder, von Willebrand disease, she declined mucogingival surgery. A diagnostic wax‐up and mock‐up was performed to establish treatment goals. The initial phase involved periodontal, orthodontic, and implant treatment. The orthodontic treatment with aligners to correct the crossbite. Subsequently, bleaching and a second mock‐up were conducted to guide prosthetic treatment. In the prosthodontic treatment, the abutment teeth were prepared using a vertical BOPT to remodel the gingival tissues, achieving the esthetic goal of repositioning the gingival margin without surgery. The provisional phase was critical for soft tissue remodeling and ensured clinical success. After stabilization of the soft tissues, a monolithic zirconia FDP was delivered, with a follow‐up of 2 years.

**Conclusions:**

A multidisciplinary treatment plan, utilizing a digital workflow, resulted in stable clinical and esthetic outcomes at the two‐year follow‐up, effectively retreating an anterior bridge using BOPT in a patient with a coagulation disorder that contraindicated complex surgical interventions.

## 1 Objective

Fixed dental prosthesis (FDP) remains a common solution for restoring damaged teeth, as well as for restoring their form, function, and esthetics, or for replacing missing teeth [1, 2]. Various tooth preparation techniques have been described for the fabrication of FDP. These finish lines can be broadly classified into two main categories: (1) horizontal finish lines, which include chamfers or shoulders; and (2) vertical finish lines, which include feather, knife‐edge, or no finish line, where the biologically oriented preparation technique (BOPT) protocol among others [3, 4, 5]. A horizontal preparation is a clearly delineated line on the tooth, determined by the clinician, and reproduced in the impression and working model. For many years, horizontal finish lines have been the preparation of choice, demonstrating good clinical survival of the restoration [6, 7, 8, 9]. Regardless of whether the preparation is vertical or horizontal preparation technique, ensuring both gingival margin adaptation and stability is crucial for fixed dental restorations [10]. Apical migration of the gingiva can result in unsatisfactory esthetics, which may be considered a major complication [3, 7, 11].

The BOPT represents a protocol that includes a specific type of vertical tooth preparation, introduced to promote long‐term periodontal tissue stability [3, 5, 12, 13, 14]. In this approach, the tooth is prepared without a finishing line, and rotary curettage of the gingival sulcus is performed simultaneously. This results in bleeding in the area, which forms a blood clot that fills the area of the supracrestal attached tissue [10, 12, 15]. Immediate placement of an interim prosthesis, 0.5–1 mm subgingivally, with a new prosthetic emergence profile, stabilizes the coagulum, which eventually matures into connective tissue. This technique can potentially reshape the gingival contour of the teeth to achieve optimal gingival architecture. Close collaboration with the dental technician is essential to ensure the success of this approach. As it is a vertical preparation without a finishing line, the new prosthetic margin can be placed in a finishing area from the gingival margin to the bottom of the gingival sulcus, as originally described by Dr. Loi [5]. This allows for a significant increase in gingival thickness and long‐term tissue stability [4, 5, 16, 17, 18].

This case report describes the replacement of an old and misfitting metal‐ceramic FDP with a monolithic zirconia FDP using BOPT and a minimally invasive approach. The treatment was conducted alongside orthodontic treatment with aligners and bleaching treatment, following a comprehensive digital workflow.

## 2 Clinical Considerations

A 56‐year‐old female patient was referred to the restorative dentistry based in the New Technologies clinic of the University Complutense of Madrid. The patient's chief complaint was the compromised esthetics appearance and food retention with her old restoration. She requested retreatment of a metal‐ceramic FDP placed 30 years ago due to dental agenesis. A diagnosis of von Willebrand disease (vWD) was revealed through the patient's medical history. This coagulation disorder, characterized by bleeding due to defects in von Willebrand factor (VWF), plays a crucial role in platelet adhesion to exposed subendothelial collagen at sites of vascular injury [19]. The most recent clinical guidelines recommend the administration of concentrates or special medications prior to dental surgery, which were considered in our treatment plan [19].

**FIGURE 1:**
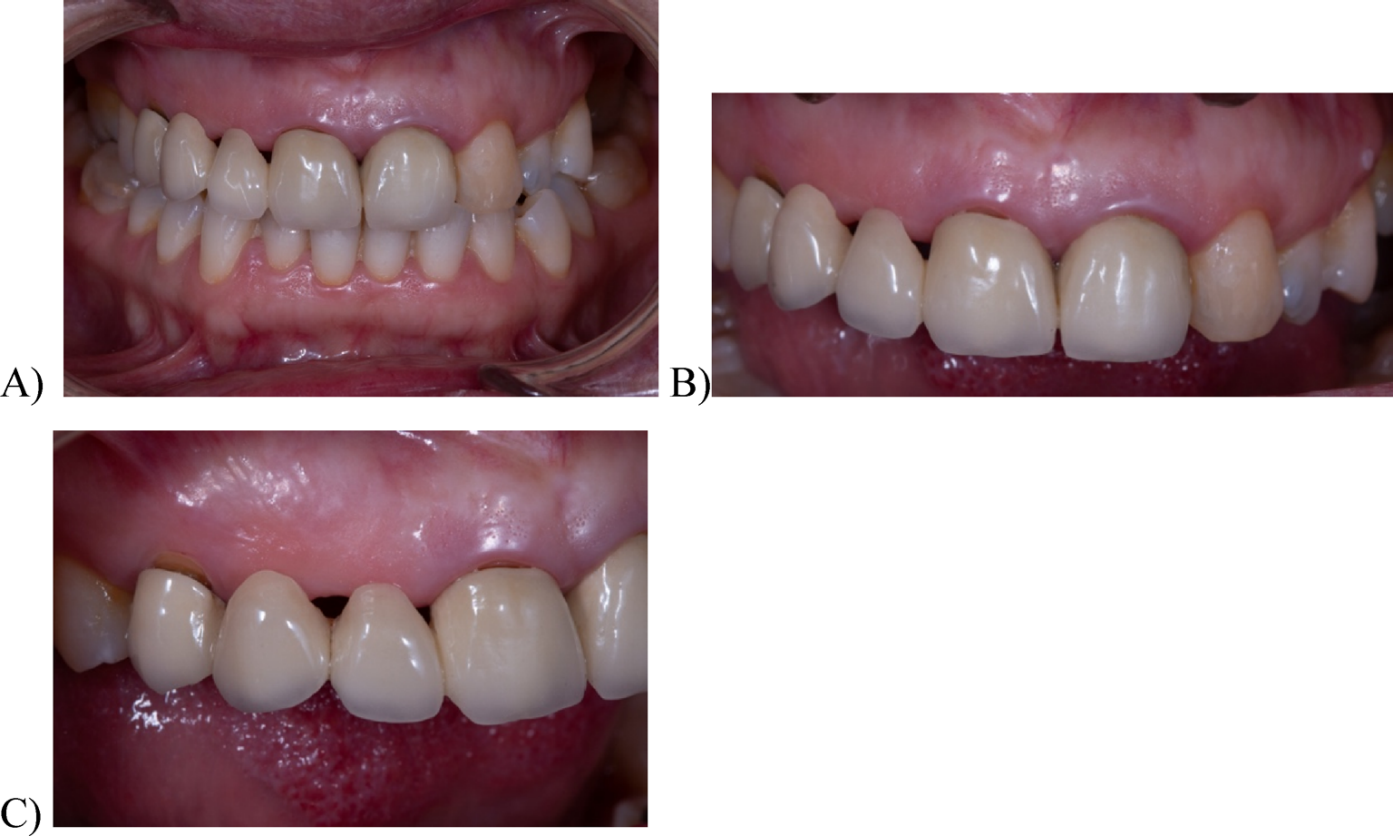
Initial clinical situation: (A) frontal photograph in occlusion; (B) initial situation of the anterior FDP in a frontal view; and (C) initial situation of the anterior FDP in a lateral view.

**FIGURE 2:**
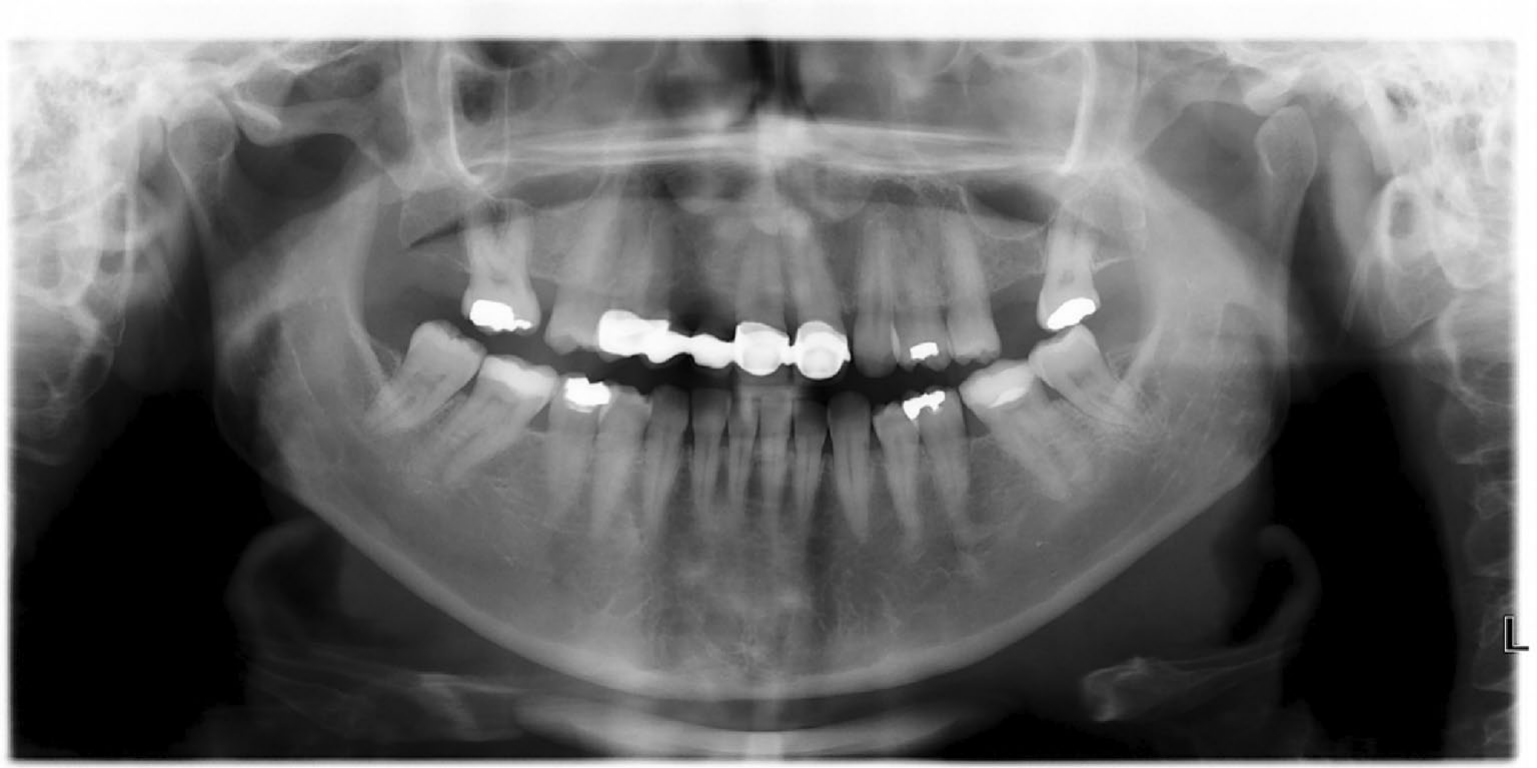
Panoramic X‐ray of the initial clinical situation.

Clinical evaluation revealed partial edentulism due to agenesia in the following teeth: mandibular and maxillary third molars, right maxillary canine and lateral incisor, and left maxillary lateral incisor. The teeth right maxillary second molar and left maxillary first molar were absent due to dental caries. An old FDP from the right maxillary first premolar to left maxillary central incisor exhibited a misfit at the gingival margin level of the abutments, with black spaces where the patient reported food retention at the pontic level, and a rounded shape of the central incisors (Figure 1). Periodontal assessment revealed localized gingivitis, with no clinical attachment loss, but with evidence of localized bleeding on probing. Radiographic examination revealed the absence of periapical or periodontal lesions (Figure 2). Occlusal analysis revealed a crossbite on the right maxillary second molar and between the second and third quadrants. Nevertheless, the patient did not report any discomfort in the head or neck nor did she experience clicking or tenderness in the temporomandibular joint (TMJ).

The treatment plan was discussed in detail with the patient, emphasizing the necessity of a multidisciplinary approach. The initial recommendation was for orthodontic treatment, supported by a preliminary wax‐up to demonstrate the need for correcting the occlusal crossbite and creatin an optimal prosthetic plan. The missing left maxillary first molar would be replaced with an implant‐supported crown, achieving an optimal occlusal plane and mesiodistal space for stable occlusion. To avoid corrective mucogingival surgery due to her coagulation condition, the plan was to replace the existing FDP with BOPT, aiming to reposition the gingival margin of the prosthesis and the edentulous ridge. A six‐month periodontal maintenance program was proposed to ensure plaque control and resolution of gingivitis before for annual maintenance. The patient consented to the proposed plan.

### 2.1 Periodontal, Orthodontic, and Implant Treatment

**FIGURE 3:**
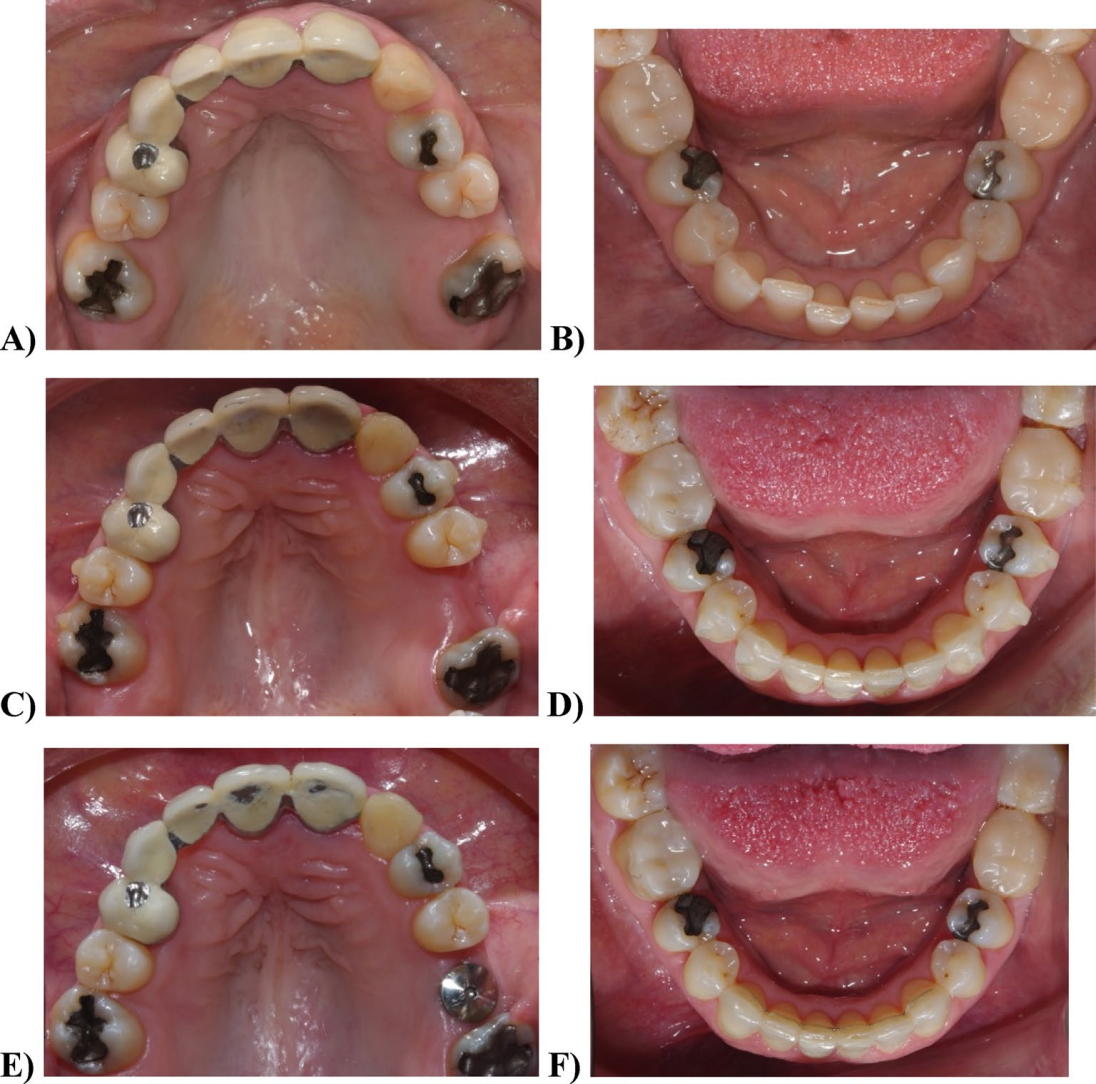
Orthodontic treatment sequence: (A) occlusal view of the maxillary diagnostic situation; (B) occlusal view of the mandibular diagnostic situation; (C) occlusal view of the maxillary jaw before the refinement treatment with clear aligners; (D) occlusal view of the mandibular jaw before the refinement treatment with clear aligners; (E) occlusal view of the maxillary final situation after orthodontic treatment and implant placement; and (F) occlusal view of the mandibular final situation after orthodontic treatment.

The treatment began with the periodontal phase, during which oral hygiene instructions were provided, and dental prophylaxis was performed to correct the gingivitis. The orthodontic phase was planned as a 12‐month‐long treatment using aligners (Invisalign Comprehensive, Align Technology, USA) to achieve the desired correction of the occlusal plane, teeth, and gingival margins alignment, and to establish optimal space for the rehabilitation of the left maxillary first molar with an implant‐supported crown (Figure 3A–F). Treatment was conducted at both the maxillary and mandibular levels to achieve stable occlusion. The teeth splinted by the previous FDP were used as reference points, as they showed no transverse occlusion alterations. After 10 months of orthodontic treatment, a 10‐millimeter (mm) mesiodistal space was obtained for an implant‐supported crown in the left maxillary first molar position (Figure 3E). A digital wax‐up was produced to guide the optimal prosthodontic position of the implant. The DICOM files obtained from a Cone Bean CT (CBCT) (Carestream, USA) were overlaid with the STL digital models (Trios 3, 3Shape, Copenhagen) using the BlueSky Plan 4 software (BlueSky Bio, USA). A 4.8 × 8 mm tissue‐level implant (WN SP, Straumann, Switzerland) was placed using a transmucosal technique without flap elevation, minimizing surgical soft tissue damage due to the patient's systemic condition. Following the completion of orthodontic treatment after 3 months, the implant achieved osseointegration. A digital impression of the implant position was obtained using a Medentika scan body (Medentika, Germany), and a monolithic zirconia crown was designed (Dental System, 3Shape, Denmark). The zirconia crown was cemented to the titanium abutment (Variobase WN, Straumann, Switzerland) with a resin cement (Multilink Hybrid Abutment, Ivoclar Vivadent, Liechtenstein) and subsequently placed, confirming the clinical and radiographic fit with a torque of 35 N.

### 2.2 Prosthodontic Treatment

**FIGURE 4:**
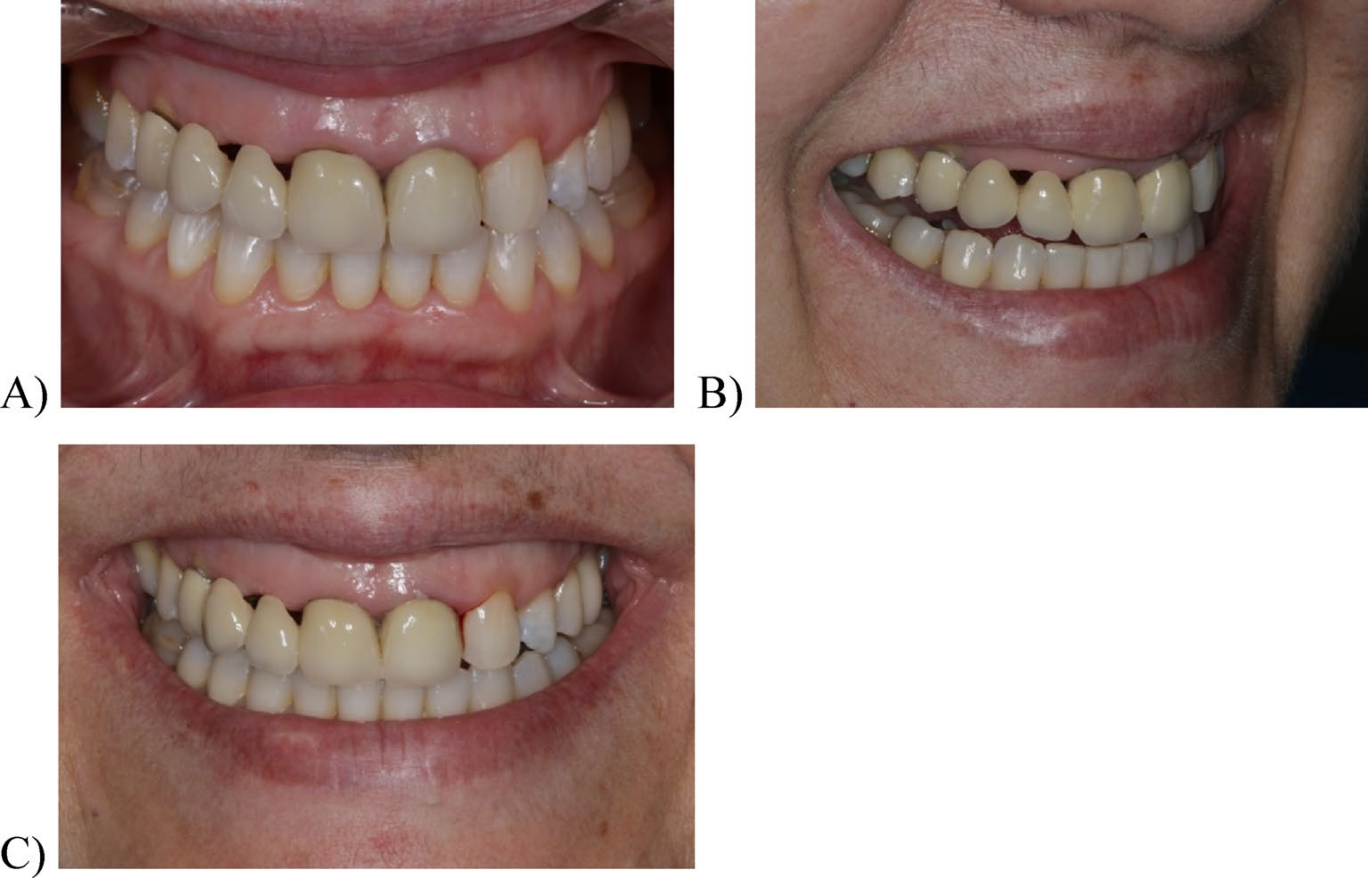
Novel prosthodontic and esthetic diagnosis after de completion of the first phase of the treatment: (A) frontal photograph in occlusion; (B) lateral view of the social smile: the patient exhibited a high smile line; and (C) frontal view of the wide and sincere smile: soft tissue exposure of ≥ 10 mm and a smile exposure of 1.6–2.6.

Upon completion of the initial treatment phase and establishment of the new occlusal position, a novel esthetic diagnosis was conducted (Figure 4A). In the lip‐resting position, 1 mm of exposure of the incisal edge of the right maxillary lateral and central incisors and 2 mm of exposure of the incisal edge of the left maxillary central incisor were observed (Figure 4B). In a social smile, the patient exhibited a high smile line with near‐complete exposure of the upper incisors and canines. In a lateral view, black spaces were still visible at the pontics level in the right maxillary lateral incisor and canine positions (Figure 4C). In a wide and sincere smile, the high smile line was confirmed, showing ≥ 10 mm of soft tissue exposure and a smile exposure from right to left maxillary with engaged buccal corridors (Figure 4D).

**FIGURE 5:**
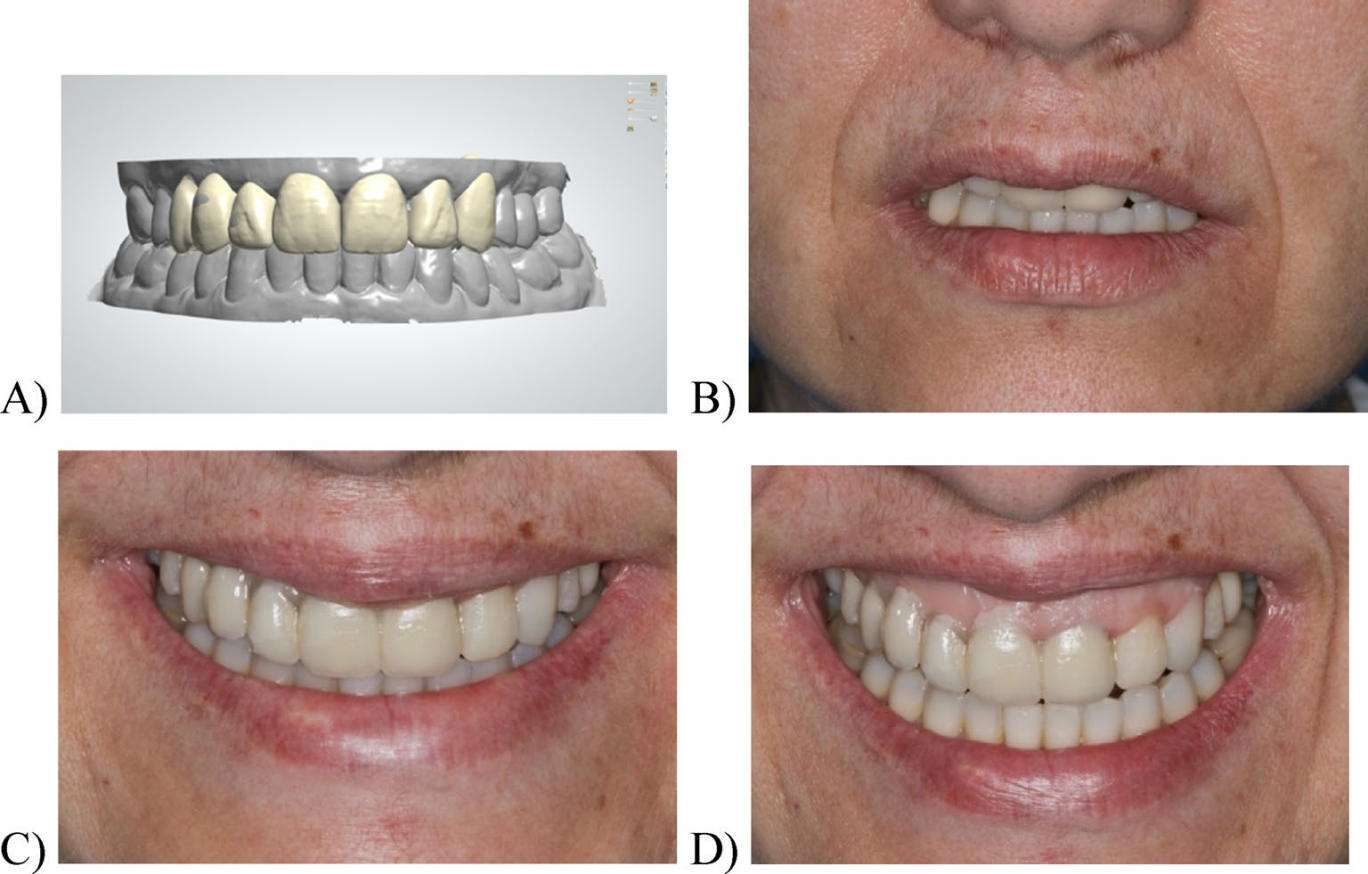
Diagnostic mock‐up with bis‐acrilic resin: (A) digital diagnostic wax‐up (dental system, 3shape, denmark); (B) lip‐resting position with the diagnostic mock‐up; (C) social smile with the diagnostic mock‐up; and (D) wide and sincere smile with the diagnostic mock‐up.

A new set of digital impressions was taken (Trios 3, 3Shape, Denmark) to create a diagnostic wax‐up (Dental System, 3Shape, Denmark), establishing new incisal edges and restorative gingival margins (Figure 4A). The digital model with the wax‐up was 3D printed (Formlabs 3B+, Formlabs, USA), and a silicone index was used to create a direct clinical mock‐up with bis‐acrylic resin (Structur, VOCO, Germany) (Figure 5B–D). The diagnostic mock‐up included the vestibular anatomical conversion, such as the 2.3 to a 2.2 and the 2.4 to a 2.3. The patient reported high levels of satisfaction with the esthetic outcome. However, she only consented to the replacement of the existing FDP restorations. The occlusal pattern was adjusted and then transferred to the intraoral scanner to create the provisional restoration.

**FIGURE 6:**
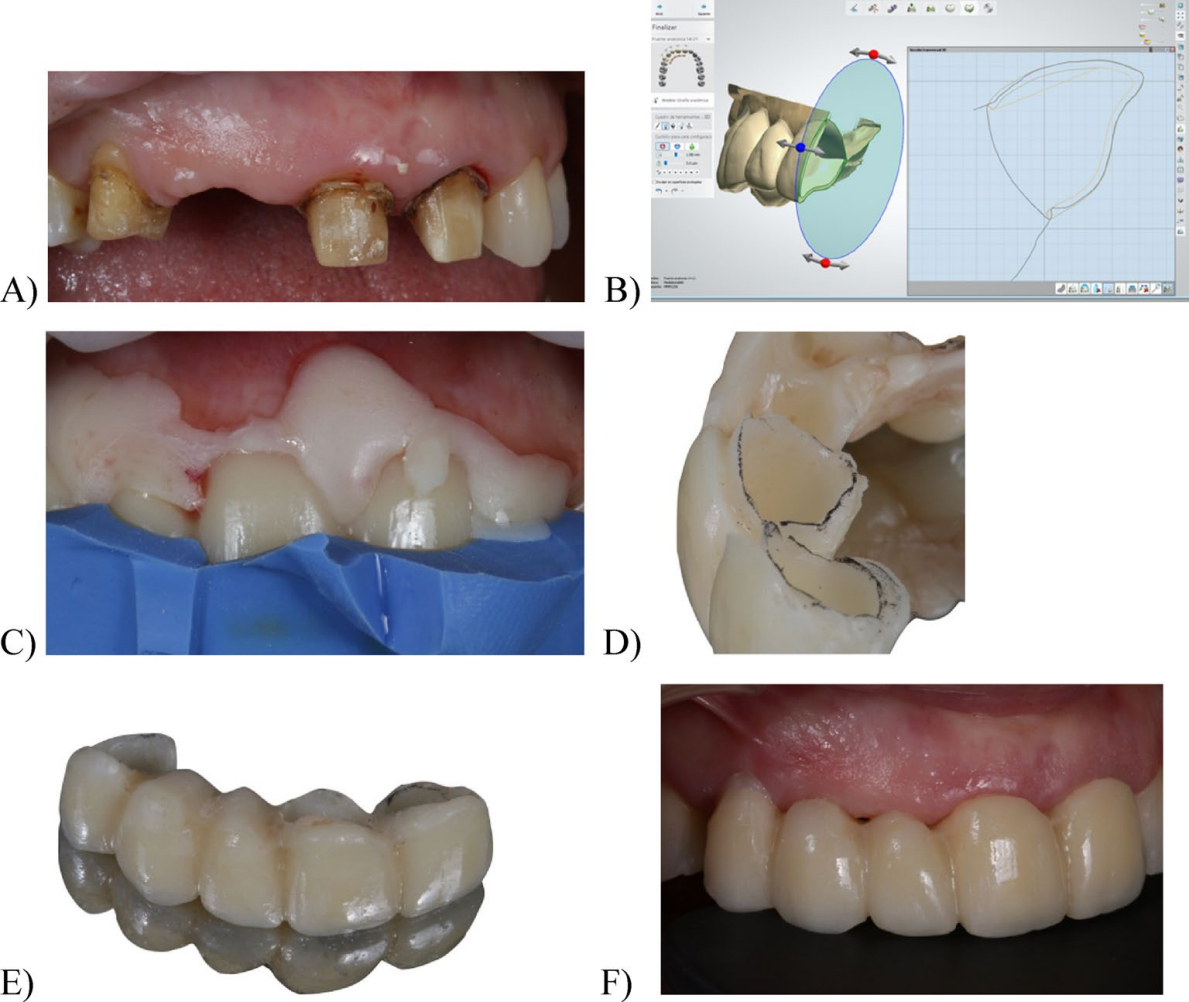
Abutments preparation and interim FDP relining following the BOPT: (A) teeth abutments after the remotion of the old metal‐ceramic restorations; (B) digital design of the hollowed milled PMMA interim FDP with a contour that follows the gingival margin; (C) silicone index to assure the optimal position during the curing and relining of the interim FDP; (D) margins reading with the interim FDP relining: the intrasulcular margin of the prepared teeth and the external margin of the gingival margin; (E) relined interim with the new gingival contour, angular component and insertion of 0.5–1 mm into the sulcus; (F) Interim FDP placed after the preparation of the abutment with the BOPT.

The previous restorations were removed using preparation burs, and periodontal probing was performed to measure the gingival sulcus depth, which determined the extent of dental preparation (Figure 6A). The purpose of BOPT was to eliminate the pre‐existing preparation of the finish line. The internal wall of the sulcus and the tooth were concurrently prepared with a conical diamond bur (BOPT burs set, Sweden&Martina, Italy) to create a smooth and refined vertical finish area, within which the crown margin could be moved coronally [5, 12]. The intrasulcular preparation involved the removal of not only the previous horizontal finish line but also the epithelial tissue and junctional epithelium of the sulcus. This procedure was carried out without significant bleeding complications.

In accordance with the diagnostic mock‐up, the dental technician had previously fabricated a hollowed, milled PMMA interim FDP with a contour that embraced the abutments 1 mm supragingivally. The interim restoration served as an impression tray for the accurate recording of the gingival sulcus following tooth preparation. (Figure 6B). Once the fit had been verified and abutments isolated with glycerin, the interim FDP was relined with acrylic resin (Sintodent C&B A2, Sintodent, Italy) and placed using a silicone index to ensure the optimal positioning during the curing phase (Figure 6C). Constant pressure was maintained with the silicone index during polymerization to acrylic resin to control any bleeding.

**FIGURE 7:**
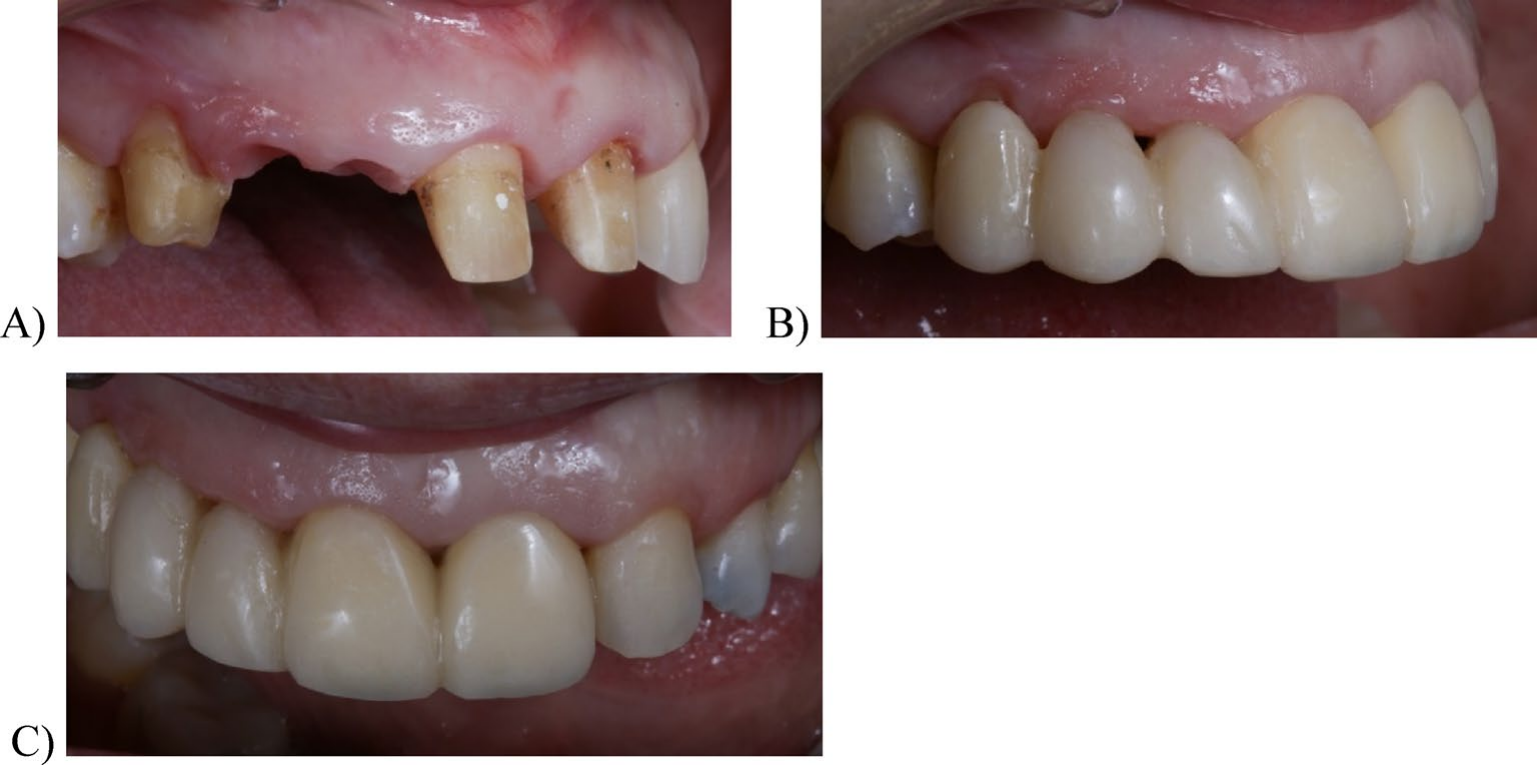
(A) Teeth abutments situation after 4 weeks of maturation of the soft tissues; (B) lateral view of the interim FDP after some morphological adjustments; and (C) frontal view of the interim FDP after some morphological adjustments.

Once the acrylic resin had set, two margins were observed: the intrasulcular margin of the prepared teeth and the external margin of the gingival margin (Figure 6D). The space between these margins was filled with fluid acrylic resin to form a new emergence for the FDP, stabilizing the soft tissue and promoting maturation of tissue with a new angular component at a depth of 0.5–1 mm into the sulcus (Figure 6E). The primary objective of the interim FDP during this phase was to stabilize and mature the blood clot formed after the abutment preparation, facilitating its eventual maturation into stable gingival tissue. The provisional profile effectively sealed the gingival margin in the desired position. On the day of preparation, the emergence profile was nearly horizontal, allowing slight pressure to stabilize the blood clot, which was particularly important given the patient's coagulation disorder. The right maxillary lateral incisor and canine were defined as ovate pontics to guide the soft tissue remodeling through targeted gingival pressure points. The interim FDP was temporarily cemented (TempBond NE, Kerr, Germany), and any excess cement material was carefully removed (Figure 6F). After a 4‐week maturation period, the provisional restoration was redefined to reduce its width, adapt the angle of emergence at the gingival margins, open space for papillae, and further shape the ovate pontics to apply pressure at the edentulous area (Figure 7A–C).

**FIGURE 8:**
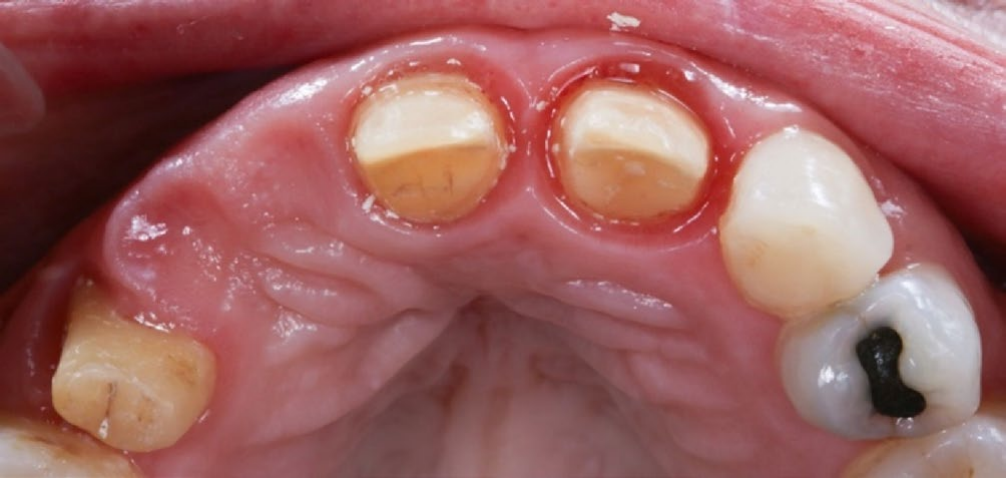
Teeth abutments and soft tissues fully matured and stable in the desired position after 10 weeks.

**FIGURE 9:**
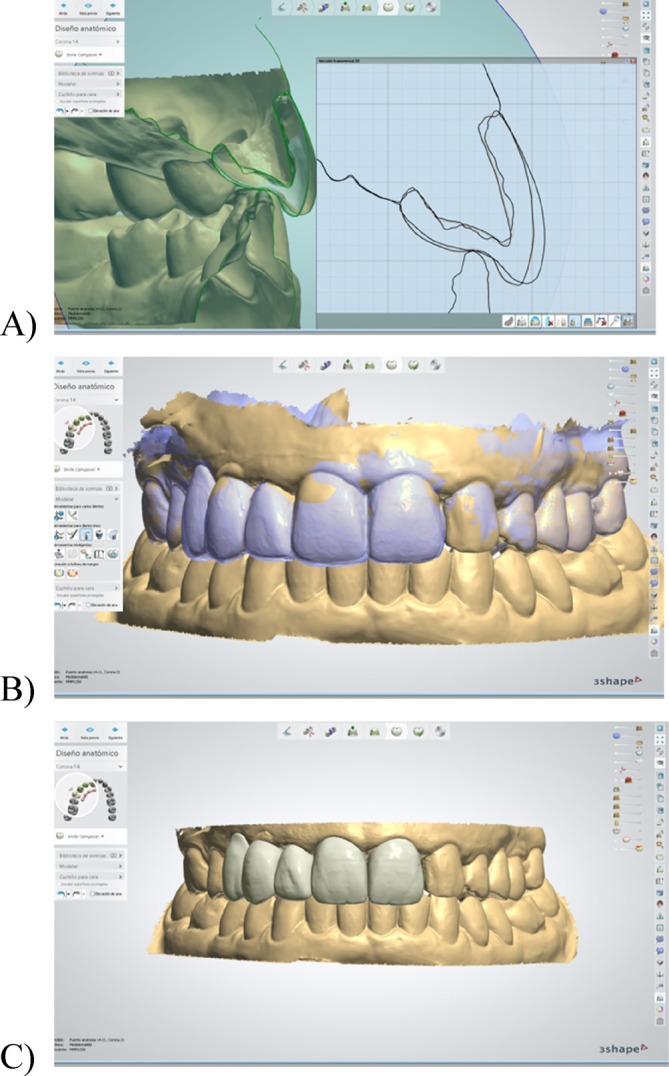
Digital design of the monolithic zirconia FDP: (A) cross‐section of the files superimposition with the diagnostic mock‐up and interim prosthesis; (B) superimposition of the diagnostic mock‐up, the interim FDP and abutments impression; and (C) digital design of the final BOPT FDP.

Final impressions were taken 10 weeks after teeth preparation, once the soft tissues had fully matured and stabilized in the desired position (Figure 8). The final digital impressions consisted of three files: (1) a digital impression of the interim FDP in place; (2) a digital impression of the abutment teeth, with special attention of capturing the internal aspect of the gingival sulcus and the finishing area; and (3) a digital impression of the interim FDP, capturing the prosthetic margin's depth and angulation. The superimposition of these digital files in the laboratory allowed the technician to identify the location of the finish area, ensuring proper placement of the finishing line for the definitive restorations and achieving an ideal prosthetic contour (Figure 9).

**FIGURE 10:**
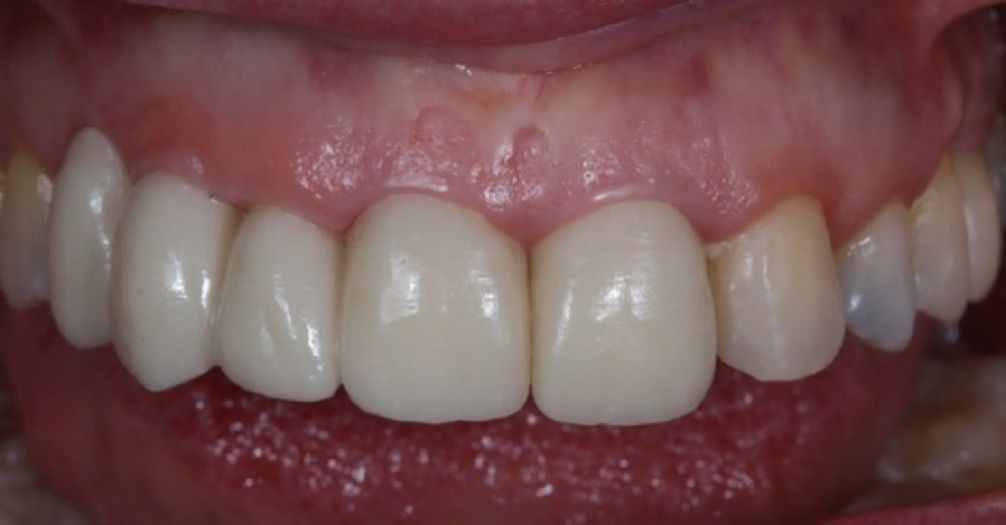
3D‐printed try‐in with temporary resin (GC Temp Print Medium, GC, Japan) of the definitive design and soft tissue adaptation.

A 3D‐printed temporary resin FDP (GC Temp Print Medium, GC, Japan) was created as a trial to check the fit, reproduction of the prosthetic margins, occlusion, and esthetic parameters with the patient (Figure 10). The definitive restoration was fabricated from monolithic zirconia (ZirCAD Prime A2, Ivoclar Vivadent, Liechtenstein) and cemented under partial isolation with dual‐curing self‐adhesive resin cement (Relyx Universal A1, 3 M, USA). Excess cement at the gingival margin was thoroughly removed both on the day of cementation and at the one‐week follow‐up. The patient was instructed in the correct oral hygiene techniques, including the use of dental floss (Superfloss, Oral‐B, USA).

**FIGURE 11:**
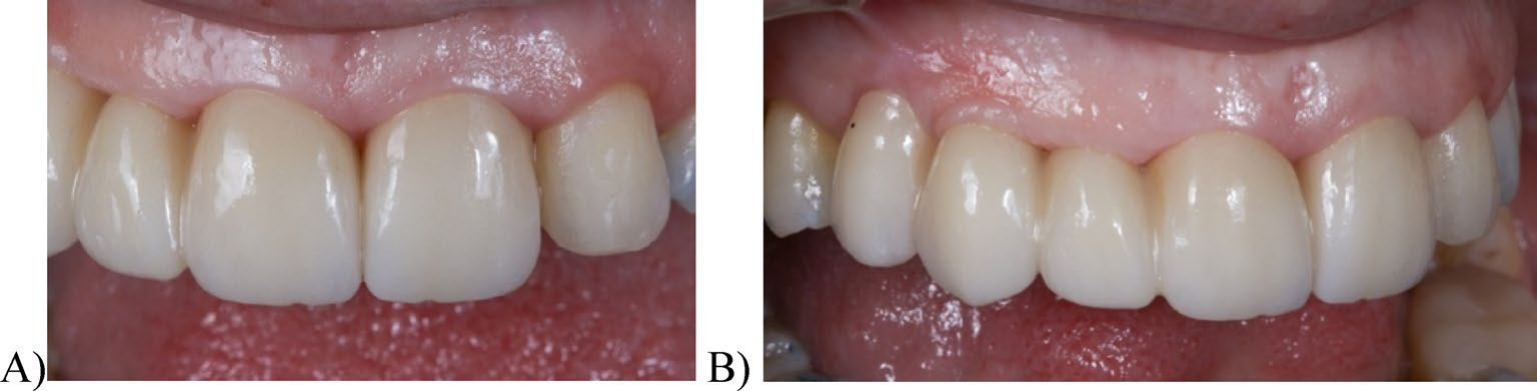
One‐week follow‐up of the definitive FDP: (A) gingival margin profiles of 1.1. and 2.1. with the definitive prosthesis; and (B) lateral view of the definitive FDP with the new soft tissue contours.

**FIGURE 12:**
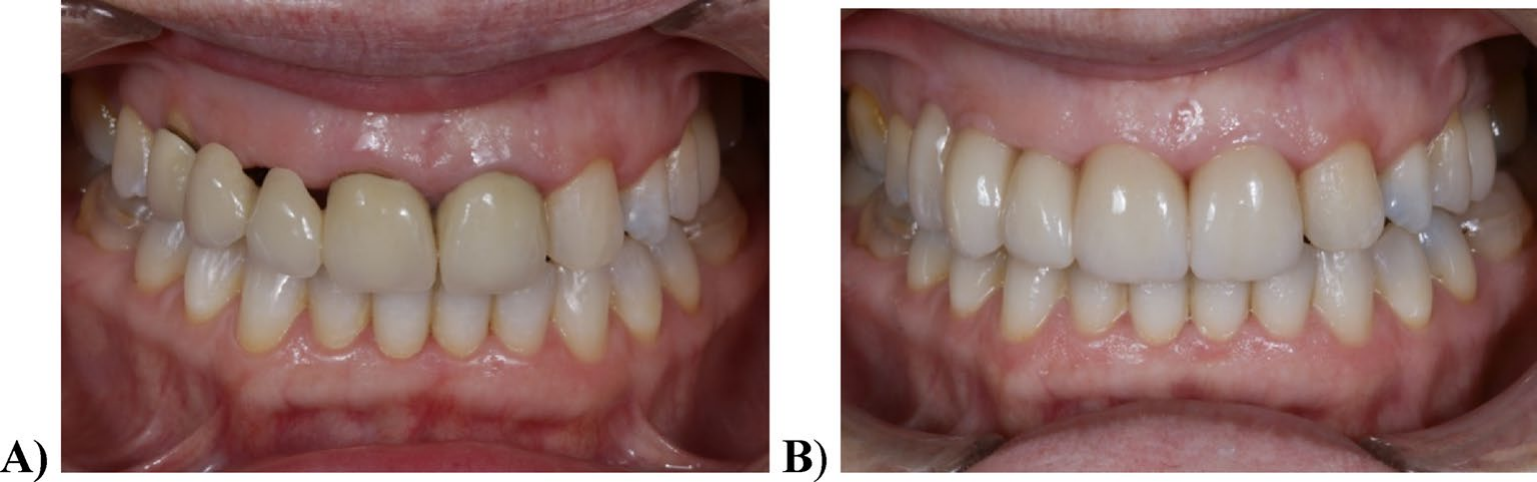
Before and two‐year follow‐up of the prosthetic treatment: (A) frontal view before the prosthetic treatment; and (B) two‐year follow‐up frontal view.

**FIGURE 13:**
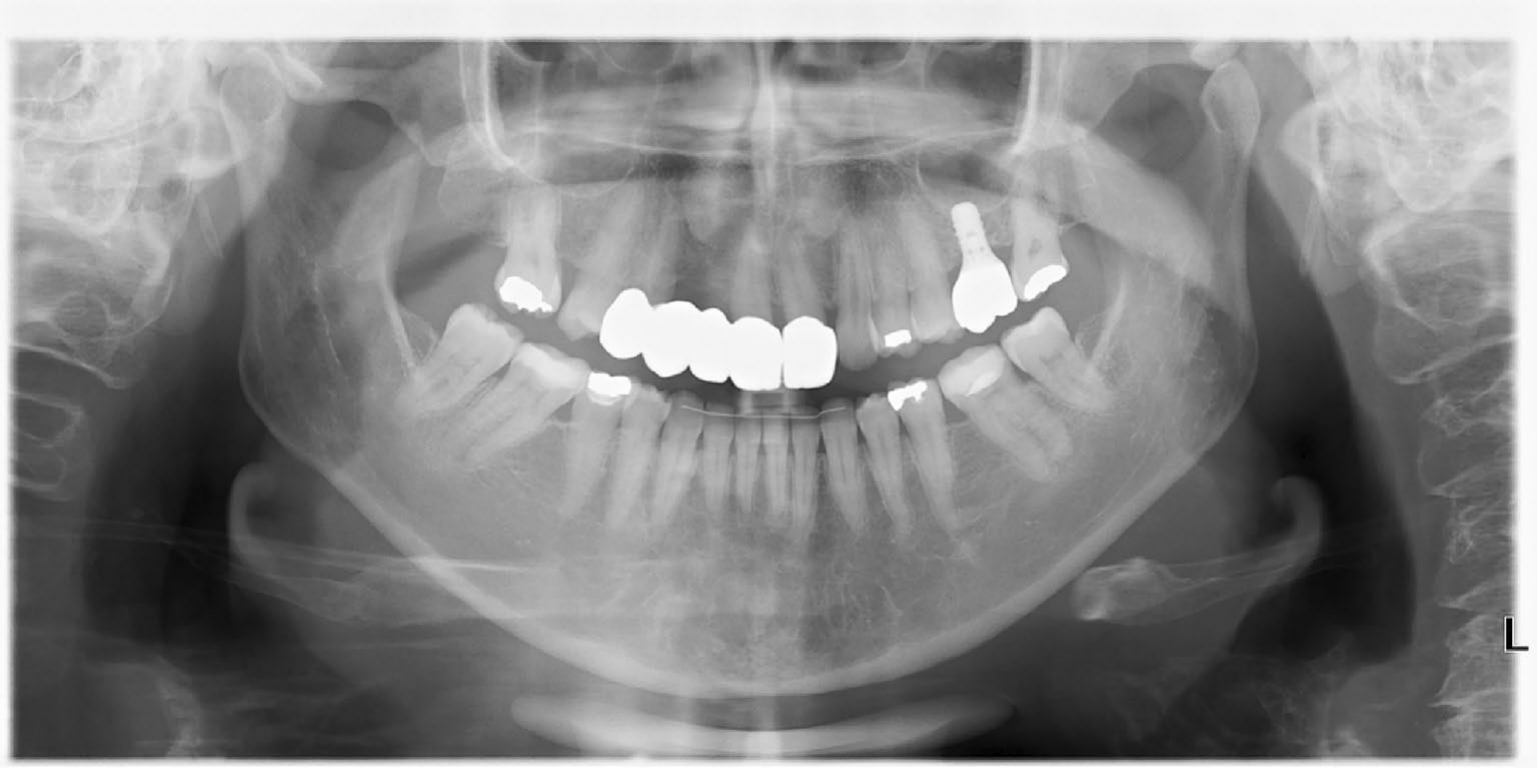
Panoramic X‐ray at the two‐year follow‐up.

At the one‐week follow‐up, the soft tissues were completely adapted to the new prosthetic contours (Figure 11A,B). The patient's esthetic expectations were met, as evidenced by the improved gingival adaptation of the new prosthesis, the closure of the black spaces at the pontic level where food retention had previously occurred, and a reduction in gingival exposure during the smile. This approach avoided the need for invasive surgical procedures. Subsequent follow‐up evaluations were conducted at 6, 12, and 24 months following the placement of the definitive prosthesis, with no mechanical, esthetic, or biological complications reported (Figures 12 and 13). Periodontal evaluation of the tooth abutments showed probing depths ranging from 1 to 3 mm.

## 3 Conclusions

The optimal method for tooth preparation remains a topic of debate within the scientific community. Both horizontal and vertical preparation lines have been associated with a range of advantages and disadvantages. The efficacy of vertical preparations has been investigated, demonstrating their effectiveness in periodontal patients with up to 20 years of follow‐up. The goal was to avoid creating horizontal finishing lines at the root level [8, 20]. Vertical preparation techniques, such as feather edge or BOPT, have been shown to improve the marginal fit of restorations and foster a favorable environment for long‐term soft tissue stability [3, 5, 11, 21, 22]. BOPT allows for the creation of a new prosthetic contour located subgingivally within the sulcus, without invading the epithelial junction, thereby guiding the soft tissues while respecting the biological width, as illustrated in this case report. This is achieved by considering esthetic parameters, such as gingival contours, the presence of the distal zenith, and the closure of the interproximal spaces [23]. This is possible due to the absence of horizontal finishing lines, which forces the emergence of the restoration at a specific level.

The preparation of the interim prosthesis to guide this tissue maturation for a minimum of 4 weeks, as originally described, has been the subject of extensive study within this technique [4, 5, 12]. However, new protocols are explored that shorten these periods, with positive results observed in posterior regions [21]. In cases with high esthetic demand, such as the case presented the role of the interim FDP is critical in guiding the gingival margins and zeniths of the teeth. Additionally, other vertical preparation techniques, performing gingitage, have been documented, showing favorable long‐term outcomes following the use of provisional restorations to control the novel prosthetic contour during the tissue maturation process [11].

The apical migration of soft tissues around the margins of crowns or FDPs has long been a significant concern in restorative dentistry. Gingival recession is associated with various etiologic factors, including gingival biotype, chronic inflammation, and inadequate prosthetic marginal fit [4, 7]. One of the primary motivations for many patients seeking prosthetic retreatment is to address esthetic concerns related to gingival recessions. Gingival recessions can be managed through mucogingival surgery, which also facilitates the management of soft tissue volume around adjacent pontics and the creation of pseudopapillae [24, 25]. In consideration of the patient's systemic condition, it was determined that invasive surgical procedures involving significant soft tissue hemorrhage, such as connective tissue grafts, should be avoided. The BOPT, when performed carefully and following accurate probing of the patient's gingival sulcus, represents a less invasive technique for achieving the desired outcomes of improved gingival emergence profiles at the abutment and pontic levels [13]. Consequently, it was decided to manage this case prosthetically, understanding that this approach would involve certain limitations in the final esthetic result, based on the analysis of soft tissue levels.

BOPT has been extensively documented in clinical cases of retreatment, where the pre‐existing preparation line is eliminated, and a new gingival insertion is formed with new prosthetic emergence profiles [4, 5, 15]. In many cases, the prosthetic treatments being replaced were metal‐ceramics FDPs or crowns, which have been shown to present higher probing depths and greater recession compared to zirconia frameworks in vertical preparations [6, 15, 21]. However, veneering zirconia frameworks with ceramic has been associated with a notable increase in marginal misfit, which is linked to an elevated risk of periodontal problems and greater microleakage [26]. It can be reasonably concluded that selecting monolithic zirconia as a restorative material in direct contact with the gingival tissue, with superior fit and tissue response, represents an optimal solution in such clinical cases [9, 21].

The vertical preparation technique without a finishing line, as described in the BOPT, has some limitations. These include the technique's inherent complexity and the additional time required for clinical implementation. It is crucial for the clinician to have precise control over the preparation technique, the handling of the interim relining phase, and the management of the prosthetic contours to ensure clinical success. Moreover, the technique requires a proper understanding of the protocol by the laboratory technician [5, 23]. Although the technique was initially described using silicone impressions and the manipulation of definitive restorations on cast models, novel digital protocols are being introduced [16, 17, 27]. Further clinical studies are required to evaluate and compare the two working methods with this specific technique, providing deeper insights into the long‐term outcomes.

In such multidisciplinary cases, long‐term follow‐up is essential for monitoring the stability of the soft tissues, occlusion, and restorations. At the two‐year follow‐up, the patient exhibited stable gingival margins, optimal integration of the dental restorations, and high satisfaction with the esthetic and functional outcomes.

Through meticulous diagnostic planning, incorporating a full digital workflow and orthodontic treatment, along with minimally invasive implant surgery and advanced restorative techniques following BOPT, a successful clinical and esthetic outcome was achieved. At the two‐year follow‐up, minimal complications were observed in the retreatment of a patient with severe gingival recession. Soft tissue management was effectively performed without the need for advance surgical intervention, due to the patient's blood coagulation disorder as a systemic condition.

## Disclosure

The authors have nothing to report.

## Conflicts of Interest

The authors declare no conflicts of interest.

## Data Availability

The data that support the findings of this study are available on request from the corresponding author. The data are not publicly available due to privacy or ethical restrictions.
